# Assessment of Medication Adherence Using a Medical App Among Patients With Multiple Sclerosis Treated With Interferon Beta-1b: Pilot Digital Observational Study (PROmyBETAapp)

**DOI:** 10.2196/14373

**Published:** 2019-07-29

**Authors:** Volker Limmroth, Klaus Hechenbichler, Christian Müller, Markus Schürks

**Affiliations:** 1 Clinic for Neurology and Palliative Medicine Municipal Hospital Köln-Merheim Cologne Germany; 2 Institut Dr. Schauerte Munich Germany; 3 Bayer Vital GmbH Leverkusen Germany

**Keywords:** digital observational study, BETACONNECT, myBETAapp, interferon beta-1b, multiple sclerosis, medication adherence, medication compliance, medication persistence

## Abstract

**Background:**

Accurate measurement of medication adherence using classical observational studies typically depends on patient self-reporting and is often costly and slow. In contrast, digital observational studies that collect data directly from the patient may pose minimal burden to patients while facilitating accurate, timely, and cost-efficient collection of real-world data. In Germany, ~80% of patients with multiple sclerosis (MS) treated with interferon beta 1b (Betaferon) use an electronic autoinjector (BETACONNECT), which automatically records every injection. Patients may also choose to use a medical app (myBETAapp) to document injection data and their well-being (using a “wellness tracker” feature).

**Objective:**

The goal of this pilot study was to establish a digital study process that allows the collection of medication usage data and to assess medication usage among patients with MS treated with interferon beta-1b who use myBETAapp.

**Methods:**

The PROmyBETAapp digital observational study was a mixed prospective and retrospective, noninterventional, cohort study conducted among users of myBETAapp in Germany (as of December 2017: registered accounts N=1334; actively used accounts N=522). Between September and December 2017, users received two invitations on their app asking them to participate. Interested patients were provided detailed information and completed an electronic consent process. Data from consenting patients’ devices were collected retrospectively starting from the first day of usage if historical data were available in the database and collected prospectively following consent attainment. In total, 6 months of data on medication usage behavior were collected along with 3 months of wellness tracker data. Descriptive statistics were used to analyze persistence, compliance, and adherence to therapy.

**Results:**

Of the 1334 registered accounts, 96 patients (7.2%) provided informed consent to participate in the study. Of these, one patient withdrew consent later. For another patient, injection data could not be recorded during the study period. Follow-up of the remaining 94 patients ended in May 2018. The mean age of participants was 46.6 years, and 50 (53%) were female. Over the 6-month study period, persistence with myBETAapp usage was 96% (90/94), mean compliance was 94% of injections completed, and adherence (persistence and ≥80% compliance) was 89% (84/94). There was no apparent difference between male and female participants and no trend across age groups. The wellness tracker was used by 21% of participants (20/94), with a mean of 3.1 entries per user.

**Conclusions:**

This study provides important information on medication usage among patients with MS treated with interferon beta-1b and on consenting behavior of patients in digital studies. In future studies, this approach may allow patients’ feedback to be rapidly implemented in existing digital solutions.

**Trial Registration:**

ClinicalTrials.gov NCT03134573; https://clinicaltrials.gov/ct2/show/NCT03134573

## Introduction

### Background

Inadequate medication usage is a major challenge in all diseases requiring long-term treatment [1]. Although adherence is typically high during the treatment of acute conditions, for chronic diseases, it decreases dramatically after the first 6 months of therapy [2]. Despite the importance of adherence to therapy, assessing medication usage in real-world settings is challenging. Large-scale retrospective studies are typically conducted using prescription data, but may not accurately measure adherence to treatment regimens [3]. Furthermore, accurate methods of measuring medication adherence using classical observational studies typically depend on patient self-reporting and are often costly and slow. In particular, longitudinal studies, needed to assess adherence over time, are more costly than cross-sectional studies or database analyses [4,5], and repeated clinic visits can be a burden for both clinicians and patients [6]. In contrast, digital observational studies that collect data directly from the patient may pose minimal burden to patients while facilitating accurate, timely, and cost-efficient collection of real-world data on medication usage.

One chronic disease for which adherence to medication is important is multiple sclerosis (MS), a chronic autoimmune disease of the central nervous system, which typically starts in young adulthood. The most common subtype of MS is relapsing–remitting MS, which is characterized by episodes of neurological dysfunction (relapses) separated by periods of remission and recovery [7]. There is no cure for MS, and effective disease management requires strict adherence to treatment regimen dose and administration schedules, typically involving disease-modifying drugs (DMDs) [8-11]. However, 25%-50% of patients with MS taking DMDs are not adherent [12,13]; among patients using injectable DMDs, the most common reason for nonadherence is forgetting to administer the injection [12]. Medication adherence among patients with MS has a direct effect on treatment outcomes, including relapse rates and health-related quality of life [8,9], as well as on health care resource utilization and costs [8-10].

**Figure 1:**
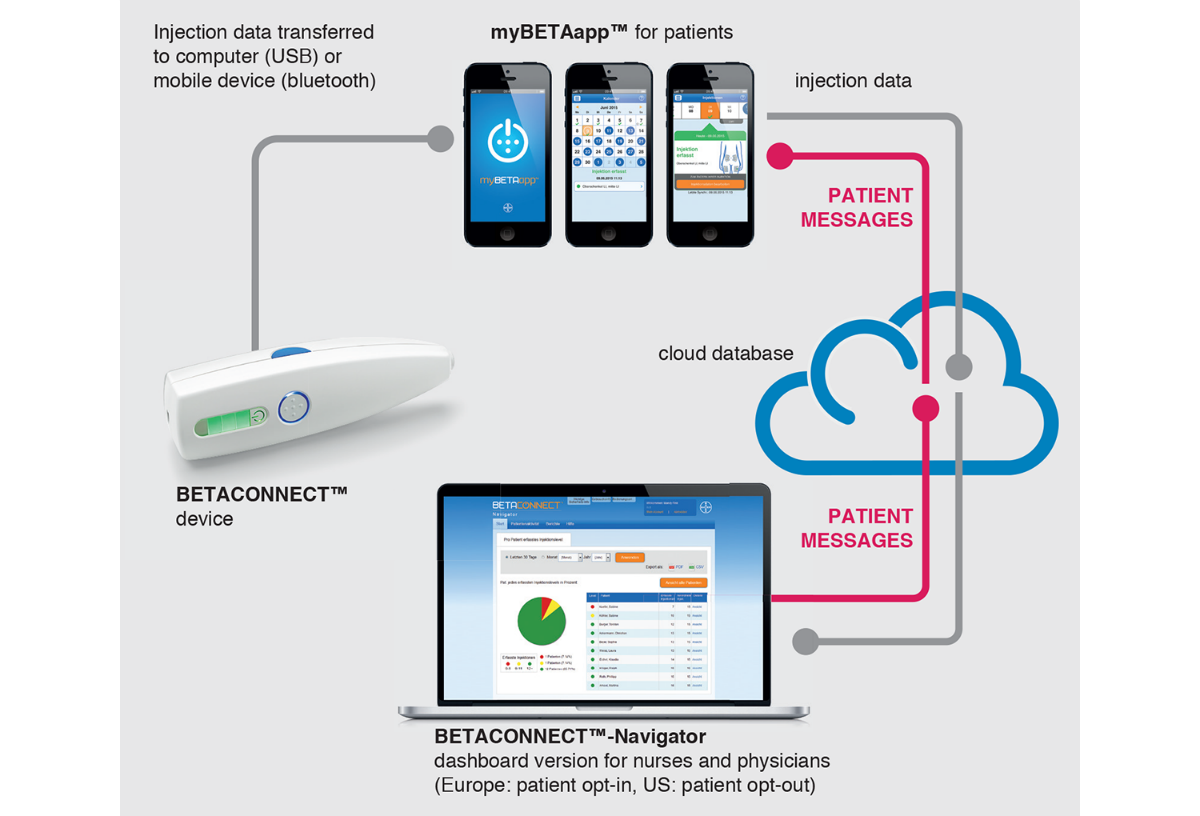
Components of the BETACONNECT system. Republished with permission from Future Medicine Ltd, from Limmroth et al, 2018 [17]; permission conveyed through Copyright Clearance Center, Inc. BETACONNECT Navigator was not part of the PROmyBETAapp study.

In Germany, ~80% of patients with MS treated with interferon beta‑1b (Betaferon) use an electronic autoinjector (BETACONNECT; Bayer, Leverkusen, Germany), which automatically records every injection. Patients may also choose to use a medical app (myBETAapp) [14] to document injection data, which can be automatically transferred from the electronic autoinjector or entered manually. Patients can also use the app to document their wellbeing using the “wellness tracker” feature, which allows them to manually record the following data on a Likert scale: ability to walk, coordination, energy level, bladder control, exercise level, memory, vision, bowel control, emotions, and eating habits. In addition, for patients who have first provided informed consent for their data to be stored in the myBETAapp database, whenever their smartphone is connected to the internet, the injection-related data and the wellness-related data are transferred to an external server under the surveillance of an external host. If patients agree (by signing a second electronic informed consent form together with their treating health care provider), data can be shared with their health care provider via an independently hosted online database (Figure 1). The three components of the BETACONNECT system—the autoinjector, myBETAapp, and the BETACONNECT Navigator—constitute an ecosystem aimed at supporting patients with MS.

Previous studies have shown that electronic autoinjector use is associated with a high level of adherence and patient satisfaction [15,16]. In addition, most patients (70/75) using myBETAapp find it helpful for regular injections, and approximately half (34/75) use the data sharing feature [17].

### Objectives

The widespread use of the electronic autoinjector in Germany makes it an ideal setting for a digital observational study. The goals of this pilot study were to establish a digital study process that allows the collection of medication usage data and to investigate medication usage among patients treated with interferon beta-1b using myBETAapp. In addition, we aimed to investigate the proportion of patients consenting to participate in the study, and the proportion willing to use the wellness tracker.

## Methods

### Study Design and Patients

The PROmyBETAapp digital observational study (trial registration: NCT03134573 [18]) was a mixed prospective and retrospective, noninterventional, cohort study conducted among users of myBETAapp in Germany (as of December 2017, registered accounts: N=1334, actively used accounts: N=522; active usage of an account is defined as having actively added, deleted, or changed data in myBETAapp during the previous month). Adult patients with MS treated with interferon beta-1b were eligible to participate in the study if they were using the app and provided electronic informed consent.

### Study Conduct and Ethics Approval

Between September and December 2017, users received two invitations on their app asking them to participate. Patients who expressed interest in the study (by pressing a button) were presented with more detailed information in a sequential manner: background, aim of the study, study design and data usage, data privacy including how to give and withdraw consent, and contact information at the database host and Bayer in case of questions. After each sequence, patients were required to confirm that they had understood the information and were finally asked to give their consent to participate. The patient informed consent document included text requesting patients to report any side effects or possible side effects to their physicians or nurses, or directly to the Bayer pharmacovigilance department. Only patients consenting to all steps were able to participate in the study.

The study protocol was approved by the ethics committee of the Nordrhein Medical Chamber (approval number AZ 2017170).

In this observational study, interferon beta-1b was prescribed in accordance with the terms of the marketing authorization. There was no assignment of a patient to a particular therapeutic strategy. The treatment decision fell within current practice, and the prescription of the medicines was clearly separated from the decision to include the patient in the study. No additional diagnostic or monitoring process was required for enrolment or during the study. Furthermore, patients made their decisions to use myBETAapp and participate in the study freely. In addition, patients were free to withdraw from the study at any time and without giving a reason; after withdrawal of a patient from the study, data from that patient were not used for any further analyses. No investigator was involved in the patient recruitment or data collection processes.

### Data Collection

Data from consenting patients’ devices were collected retrospectively, starting from the first day of usage if historical data were available in the database and collected prospectively following consent attainment. In total, 6 months of data on medication usage behavior were collected along with 3 months of wellness tracker data. With respect to the wellness tracker, the only data recorded were whether a participant used it and, if so, how often. All data collected in myBETAapp were transferred via the internet to a database hosted by TWT Digital Health GmbH (Heidelberg, Germany). Data collected directly or calculated by TWT Digital Health GmbH based on directly collected data are listed in Table 1.

**Table 1:** Data derived from the electronic autoinjector and myBETAapp (directly collected or calculated) that were transferred to the study database.

Data		Description
**BETACONNECT injection**		
	patient_id	Patient identifier
	injection_date_timestamp_utc	Data recorded via BETACONNECT: date and time of injection within the first 6 months of usage
	dose	Data recorded via BETACONNECT: dose of interferon beta-1b; possible values: 0.25, 0.5, 0.75, 1.00
	injection_site	Data recorded via BETACONNECT: “unknown”; manual data entry via myBETAapp
	flag_manual_autoinjector	Autoinjector: data recorded via BETACONNECT; Manual: data manually entered by patient into myBETAapp
	needle_depth	Data recorded via BETACONNECT: injection depth; possible values: 8, 10, 12
	injection_speed	Data recorded via BETACONNECT: speed of injection; possible values: low, medium, high
**Patient**		
	patient_id	Patient identifier
	acceptance_date_utc	Date of patient’s consent to participate
	first_injection_date_utc	Not used for analysis
	age	Age of patient
	gender	Gender of patient; possible values: M, F
	complete_wellness_number	Number of completed wellness tracker entries within the first 3 months of usage

### Data Quality

Data stored in the database were cleaned before analysis. Patients were free to choose not to download their injection data from the electronic autoinjector into the app, instead documenting injections manually or choosing not to record any injection information in the app. In addition, a number of factors could have affected the quality of the collected data, including data collection and synchronization of data from the electronic autoinjector at multiple time points, patient preferences for the myBETAapp reminder function, and settings for forwarding data from the app to the database. As a result, it was possible for the database to include multiple injection records for a given day. Therefore, prior to data analysis injection, data were automatically corrected according to the rules described in Textbox 1. Redundant data not considered by these rules were not cleaned manually. All data transferred to Bayer were anonymized.

**Data cleaning procedure.**

**Identical data entries**

- Duplicate data entries were deleted.

**Multiple data entries on same day**

- If more than one autoinjector record or more than one manual data entry was available for the same day, the last entry (ordered by time) was kept.

**Combination of manual and autoinjector data on the same day**

- If both autoinjector and manual data entries were present for a given day, the autoinjector record was used.
- If the autoinjector record injection site was “unknown,” the injection site from the manual record was substituted.
- If the autoinjector record included injection site data, the autoinjector record was used, even if the manual record included different injection site data.

### Statistical Analysis

#### Statistical Methods

Analyses of persistence, compliance, and adherence, defined as shown in Textbox 2, were conducted using descriptive statistics. The analyses included a stratified analysis according to gender and age subgroups.

Missing data can result from various reasons, including technical issues and patients choosing not to document additional information on injections via the app.

**Study definitions.**

- Persistence: The patient was still using interferon beta-1b at the end of the 6‑month study period.
- Compliance: Percentage of expected doses actually injected.
- Adherence: The patient was both persistent and ≥80% compliant.

#### Study Size

Injection-related data from 40 patients would allow determination of mean compliance (%) with ±10% for a two-sided approximate 95% CI with >99% confidence (assuming an SD of 15%-20%).

### Sensitivity Analysis

Sensitivity analyses were conducted on data derived from the BETACONNECT autoinjector only. A patient was included in the sensitivity analysis if he/she had provided only injection data recorded by the BETACONNECT throughout the observation period.

## Results

### Participants

Of the 1334 registered myBETAapp accounts, 96 patients (7.2%) provided their informed consent to participate in the study. Of these, one patient withdrew informed consent later. For another patient injection data could not be recorded during the study period, apparently because of a technical issue. Follow-up of the remaining 94 patients ended in May 2018. The mean age of participants was 46.6 years, 50 (53%) were female, and 44 (47%) were male (Table 2).

**Table 2:** Study participants according to their age (N=94).

Age group (years)	Total, n (%)	Female, n (%)	Male, n (%)
<30	10 (11)	9 (18)	1 (2)
30-39	23 (24)	14 (28)	9 (20)
40-49	32 (34)	15 (30)	17 (39)
50-59	15 (16)	5 (10)	10 (23)
≥60	14 (15)	7 (14)	7 (16)

### Data Collected

#### Injection Data

For most patients (54/94, 57%), only autoinjector data were available, while 26 of 94 patients (28%) used both autoinjector data and manual documentation (Table 3). In total, 31 of the 50 female participants (62%) and 23 of the 44 male participants (52%) did not enter any manual data. In the group aged 30-39 years, only 8 of the 23 patients (35%) used autoinjector data alone, and 11 (48%) used both autoinjector data and manual documentation. The majority of participants (60/94, 64%) recorded injection location data.

**Table 3:** Injection and wellness tracker data collected (N=94).

Data				Total	Female	Male
**Injection, n (%)**						
	**Injection patterns**					
		Autoinjector data only	54 (57)		31 (62)	23 (52)
		Manual data only	14 (15)		8 (16)	6 (14)
		Both autoinjector and manual data	26 (28)		11 (22)	15 (34)
	**Injection location data**					
		Yes	60 (64)		31 (62)	29 (66)
		No	34 (36)		19 (38)	15 (34)
**Wellness tracker**						
	**Use of wellness tracker, n (%)**					
		Yes	20 (21)		13 (26)	7 (16)
		No	74 (79)		37 (74)	37 (84)
	**Number of wellness tracker entries per user**					
		Number of users	20		13	7
		Mean (SD)	3.1 (4.1)		3.6 (5.1)	2 (0.8)
		Minimum	1		1	1
		Q1^a^	1		1	1
		Median	2		1	2
		Q3^b^	2.5		2	3
		Maximum	17		17	3

^a^Q1: lower 25% quartile.

^b^Q3: upper 75% quartile.

#### Wellness Tracker Data

Over the 3-month observation period, the wellness tracker was used by 20 of 94 participants (21%), with a mean of 3.1 entries per user (Table 3). Female patients were more likely to use the wellness tracker than male participants (13/50, 26%, vs 7/44, 16%) and made more entries (mean: 3.6 vs 2.0 entries per user).

### Medication Usage

**Figure 2:**
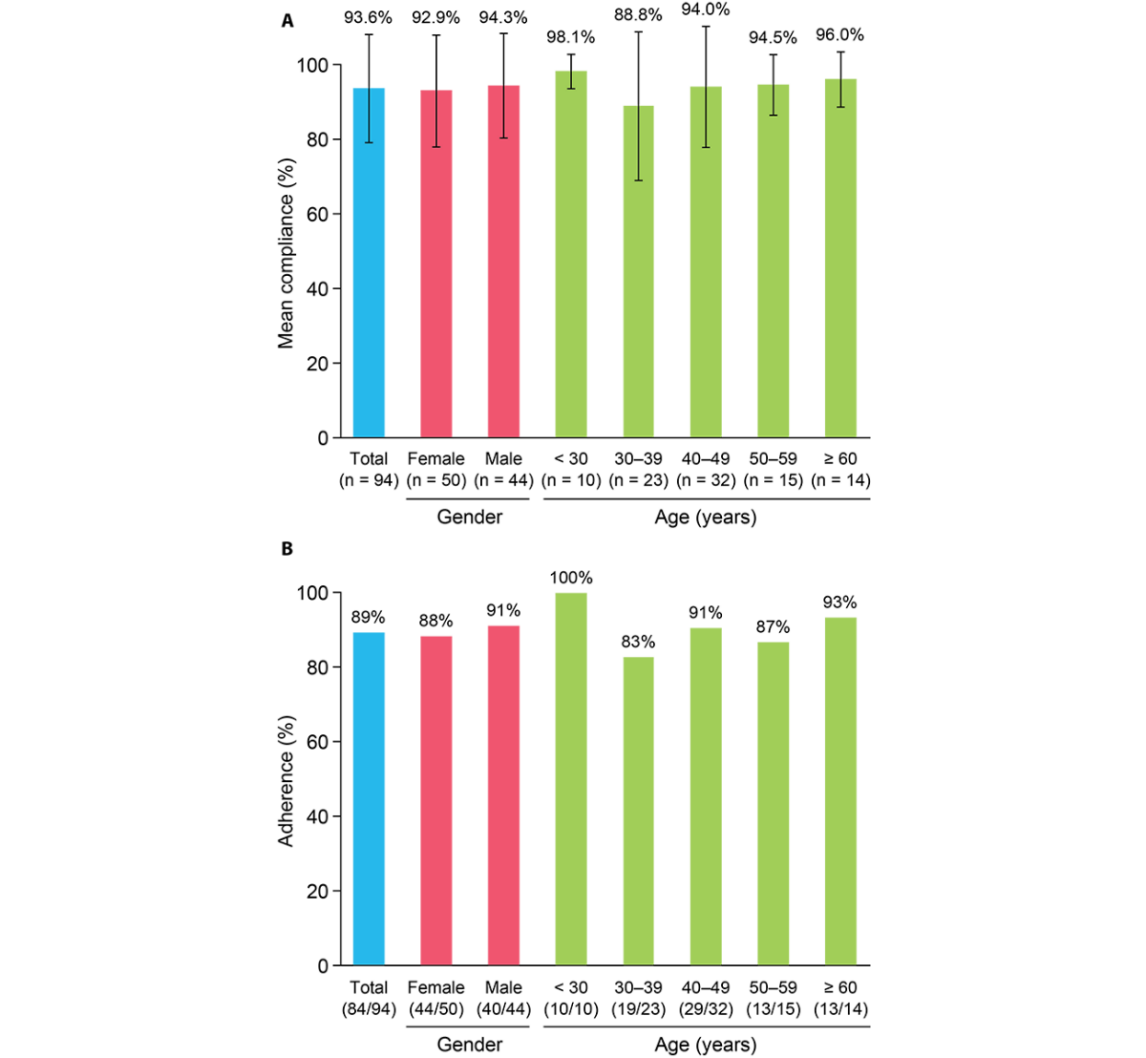
(A) Mean compliance and (B) adherence over the 6-month study period. Bars in (A) indicate SD.

Over the 6-month study period, persistence was 96%, with only 4 of 94 patients discontinuing treatment. The majority of patients did not miss any injections (median compliance: 100%, mean: 94%, Table 4 and Figure 2). Compliance was lowest among patients aged 30-39 years (median 100%, mean 89%). Adherence was 89% over the 6‑month observation period (84/94 participants were adherent; Figure 2). There was no apparent difference between female (44/40, 88%) and male (40/44, 91%) participants and no trend across age groups.

**Table 4:** Persistence, compliance, and adherence in the main analysis and sensitivity analysis groups.

Data				Total		Female		Male
**Main analysis group**								
	Participants, n (%)			94 (100)		50 (53)		44 (47)
	**Persistence, n (%)**							
		Yes	90 (96)		47 (94)		43 (98)	
		No	4 (4)		3 (6)		1 (2)	
	**Compliance**							
		Mean (SD)	93.6 (14.5)		92.9 (15)		94.3 (14)	
		Minimum	23.3		35.6		23.3	
		Q1^a^	94.4		94.4		95.6	
		Median	100		99.4		100	
		Q3^b^	100		100		100	
		Maximum	106.7		106.7		106.7	
	**Adherence, n (%)**							
		Yes	84 (89)		44 (88)		40 (91)	
		No	10 (11)		6 (12)		4 (9)	
**Sensitivity analysis group**								
	Participants, n (%)			54 (100)		31 (57)		23 (43)
	**Persistence, n (%)**							
		Yes	52 (96)		29 (94)		23 (100)	
		No	2 (4)		2 (6)		0 (0)	
	**Compliance**							
		Mean (SD)	93.6 (13.6)		92.8 (15.7)		94.7 (10.5)	
		Minimum	35.6		35.6		57.8	
		Q1^a^	95.6		95.6		94.4	
		Median	98.9		98.9		100	
		Q3^b^	100		100		100	
		Maximum	101.1		101.1		101.1	
	**Adherence, n (%)**							
		Yes	49 (91)		28 (90)		21 (91)	
		No	5 (9)		3 (10)		2 (9)	

^a^Q1: lower 25% quartile.

^b^Q3: upper 75% quartile.

### Sensitivity Analysis

All analyses performed for the full analysis set (N=94) were repeated in the subgroup of patients for whom only autoinjector data were available (N=54; Table 4). The demographics of the sensitivity analysis group (mean age, 47.2 years; 31/54 or 57% female) were similar to those of the full analysis set (Table 2). Persistence (96% in both analyses [90/94 and 52/54 participants included in the full analysis and sensitivity analysis, respectively]), compliance (median: 100%, mean: 94% in full analysis; median: 99%, mean: 94% in sensitivity analysis), and adherence (84/94, 89% in full analysis vs 49/54, 91% in sensitivity analysis) were similar in the two groups.

## Discussion

### Principal Results

This pilot study demonstrates the feasibility of a digital observational study design, with recruitment, consent, and data collection conducted via myBETAapp, and data retrieved and processed in a time- and cost-efficient manner. The proportion of patients participating was 7.2% of the registered accounts. Injection data were successfully obtained for almost all participants, and the sensitivity analysis showed that useful data can be generated without any manual input from patients. Only a minority of patients chose to use the optional wellness tracker function. The results show that for patients with MS using the app in Germany, persistence, compliance, and adherence over the 6-month period were high.

### Comparison With Prior Work

Overall, the proportion of participants who were male (44/94, 47%) was higher than that seen in a recent German observational study of patients with MS using interferons or glatiramer acetate (120/429, 28%) [19]. This proportional shift may be due to the digital study design.

Adherence based on data collected through myBETAapp was slightly higher (84/94, 89%) than that obtained in a previous nondigital study of patients using the electronic autoinjector (62/77, 80.5% [at 24 weeks]) [15]. A potential explanation for this may be the reminder function implemented in myBETAapp; however, we did not investigate use of this feature in our study. No comparative data on persistence, compliance, or adherence between patients receiving interferon beta-1b who use the myBETAapp and those who do not use the app are available. A similar proportion of adherent patients (131/158, 82.9% [at 24 weeks]) was seen in another nondigital observational study of patients with MS using a different electronic injection system [20]. Therefore, medication usage data obtained through the app are likely to be reliable.

Consistent with previous studies on MS [8,21,22], in this analysis, compliance and adherence to medication were lowest among participants aged 30-39 years. For this group of working-age patients, the effectiveness of MS treatment may have a significant impact on long-term health-related quality of life. One strength of digital observational studies of this kind is the potential to rapidly distinguish patient cohorts for whom particular treatment regimens or support may be appropriate.

### Potential of Digital Observational Studies

Compared with classical observational approaches, digital studies have considerable potential to reduce costs and improve efficiency. The costs of conducting longitudinal observational studies can approach those of randomized controlled trials [5], primarily due to the costs associated with clinical sites. In particular, recruitment of a large patient population may necessitate the involvement of many clinical sites, leading to complexity and high costs; in the United States, site management costs make up more than half the cost of phase 4 trials [23]. To avoid site costs, a UK trial of 15,480 patients with diabetes (ASCEND) was conducted by mail, with overall costs an order of magnitude lower than those of traditional clinical studies [24]. However, recruitment in the ASCEND trial was slow [24], a common problem in clinical studies [25]. By enabling eligible patients to be approached rapidly outside of routine clinic visits, digital methods may enable studies to be completed more quickly than traditional studies and at a lower cost. In addition, once the digital platform has been established, the marginal cost of adding additional patients is small, meaning that digital observational studies can potentially enroll very large populations of patients. For example, the Apple Heart Study recently enrolled more than 400,000 participants in a 9-month period [26].

Many patients are happy to take part in clinical studies, both to potentially improve their own treatment and help others by contributing to scientific research [27]. However, participation may be limited for several reasons, including the inconvenience of having to attend additional clinic visits [28]. In addition, patients whose doctors are not investigators in clinical studies may not be offered the opportunity to participate [29]. Both of these barriers to clinical study participation can potentially be overcome by digital study designs, with patients being able to take part in studies remotely and potentially without the direct involvement of their treating physician. Appropriately designed digital recruitment processes may also increase patient engagement; directly approaching patients to participate may make them feel like their contribution is valued and important in a way that being selected by their physician would not. The US Food and Drug Administration’s newly developed MyStudies app is an example of a “platform” app that may facilitate the conduct of digital studies on a large scale [30].

### Limitations

This pilot study has several limitations. First, the data used in this study were obtained both prospectively (after informed consent) and retrospectively (before informed consent). Although retrospectively collected data in observational studies are prone to recall bias, we believe that this is not an issue in our study. Although some data were collected before the start of the study, they were collected in the same way as the prospective data, by automatic recording of injection data. However, we cannot exclude the possibility that participation in the PROmyBETAapp study may have changed the injection behavior of the participants. Second, the technology used to obtain data on medication intake behavior using the electronic autoinjector and myBETAapp has not been validated. However, there is no gold standard for recording medication intake in observational studies, and direct surveillance is not feasible in such a setting. Third, technical issues with the smartphone (eg, connection between the electronic autoinjector and the smartphone, or between the smartphone and the server) as well as patients’ decision not to document additional data may have led to missing data, which have not been replaced. Fourth, for the 4% of patients who were classified as nonpersistent, it is not possible to distinguish between patients discontinuing treatment and those simply ceasing to document their injections in the app. Fifth, only patients using interferon beta-1b participated in the study, limiting generalizability to patients using other DMDs. However, the aim of the study was to investigate medication intake behavior among patients using the app, and the electronic autoinjector is only available to patients using interferon beta-1b and not to those using other medications. Sixth, only 7.2% of the registered account users consented to participate in the study, which may further limit generalizability to all patients using interferon beta-1b. Seventh, it is possible that mainly technophile patients who were using myBETAapp decided to participate in the study, which may constitute a selection bias. However, MS predominantly affects young people, who tend to be familiar with using mobile devices and apps, and any bias introduced is likely to be limited. In addition, in this pilot study, the results for persistence, compliance, and adherence were similar among men and women and across age groups. Eighth, the results must be interpreted with caution due to the limited sample size, especially in the gender and age subgroups. Finally, results beyond 6 months are not yet available. However, studies using the same design over longer periods of time are planned.

### Conclusions

This study provides important information on medication usage and consenting behavior of patients in digital studies. Persistence, compliance, and adherence over a 6-month period were high for patients with MS using the app. There are some open questions, mainly regarding recruitment and study conduct, which need to be addressed in the future studies. For example, we need to develop more refined approaches to ensure that participating patients are representative of the whole population of interest. In this context, data privacy aspects may be important. Specifically, data privacy may determine patients’ willingness to share data, in general (ie, to participate or not participate in a digital study). Willingness to share certain data may further differ by data type, with clinical and wellness data being potentially more sensitive than medication usage data. In addition, we need to use methodology that allows us to differentiate between patients terminating their medication and those simply terminating use of myBETAapp. In future studies, this approach may allow patients’ feedback to be rapidly implemented into existing digital solutions. More comprehensive studies using the digital observational design will be conducted, investigating more clinical and patient-reported outcomes.

## Abbreviations

DMD: disease-modifying drug
MS: multiple sclerosis
